# Disseminated Nocardiosis: A Case Report

**DOI:** 10.7759/cureus.5294

**Published:** 2019-08-01

**Authors:** Ines M Leite, Frederico Trigueiros, André M Martins, Marina Fonseca, Tiago Marques

**Affiliations:** 1 Serviço De Medicina 2, Hospital De Santa Maria, Lisboa, PRT; 2 Serviço De Doenças Infecciosas, Hospital De Santa Maria, Lisboa, PRT

**Keywords:** nocardia, nocardia infection, immunosuppression

## Abstract

Disseminated nocardiosis is a rare infection associated with underlying immunosuppression, and patients usually have some identifiable risk factor affecting cellular immunity. Due to advances in taxonomy and microbiology identification methods, infections by *Nocardia *species are more frequent, making the discussion of its approach and choice of antibiotherapy increasingly relevant.

A 77-year-old man presented to the emergency department with marked pain on the right lower limb, weakness, and upper leg edema. He had been diagnosed with organized cryptogenic pneumonia one year before and was chronically immunosuppressed with methylprednisolone 32 mg/day. Blood cultures isolated* Nocardia cyriacigeorgica*. Computed tomography revealed a gas collection in the region of the right iliacus muscle with involvement of the gluteal and obturator muscles upwardly and on the supragenicular plane inferiorly. Triple therapy with imipenem, amikacin, and cotrimoxazole was started, and the patient was submitted for emergent surgical decompression, fasciotomy, and drainage due to acute compartment syndrome. The patient had a good outcome and was discharged from the hospital after 30 days of intravenous therapy. This case illustrates the severity of Nocardia infection and highlights the need for a meticulous approach in the diagnosis and treatment of these patients.

## Introduction

In the suborder of Corynebacterineae, three genera have strains that may be pathological to humans, with some characteristics similar to Fungi: *Mycobacterium*, *Corynebacterium*, and *Nocardia*. In general, these are rod-like, gram-positive, ubiquitous, and aerobic bacteria with a complex cell wall and high G:C content in their DNA [1-3].

The genus *Nocardia* differs from the other genera due to the quantity of the mycolic acid content on the cell wall. In recent years, with new molecular techniques, *Nocardia* has been reclassified with an increasing number of species being recognized as human pathogens. Also, the effectiveness of sulfonamides as the first-line antibiotherapy has been questioned [1,4].

The genus was first discovered by Edmond Nocard, a French veterinarian, in 1888-1889 [5,6]. The different species are saprophytic bacteria, commonly found in soil, water, and plants, especially in areas with abundant organic residues. *Nocardia* infections in humans can be local or disseminated and are associated with underlying immunosuppression; *Nocardia* infections are rare in healthy individuals [4]. Nevertheless, cases of infection in immunocompetent subjects have been reported, especially in individuals with comorbidities [7].

If the integrity of the physical barrier of skin and mucosa is compromised, *Nocardia* species can gain entry. Once inside, both unspecific inflammatory macrophage activation and specific production of immunoglobulins G, A, and M are detected. The key element in the host’s response is cellular immunity: T lymphocytes stimulate the reticuloendothelial system against the infection, and some populations of T lymphocytes destroy the bacteria directly [6].

In most cases of infection by *Nocardia* species, patients have some identifiable risk factors affecting cellular immunity. Among those risk factors, corticosteroid therapy is considered the most important followed by human immunodeficiency virus infection, solid organ transplant, cancer, chronic pulmonary disease, and autoimmune disease [4,8]. Corticosteroids have profound effects on the cellular functions of leukocytes, impairing their entry into infection sites, reducing the clearance of bacteria by the reticuloendothelial system, causing lymphopenia and redistribution of lymphocytes to other sites, and affecting T cells more than B cells. On T cells, the effects of low-dose glucocorticoids are pleomorphic and different for each subset. Naïve CD4+ T cells are affected more than mature CD4+ effector and memory subsets, Th17+ T cells, and CD8+ effector T cells; levels of T regulatory cells are not significantly affected. At higher doses, glucocorticoids produce a rapid depletion of most circulating T cells [9,10].

We present a case of disseminated nocardiosis in an immunocompromised patient due to corticosteroid therapy.

## Case presentation

A 77-year-old man was admitted to the infirmary with erysipelas of the right lower limb and diagnosed with deep venous thrombosis on the same limb.

The patient had been admitted to the Pneumology ward the year before, diagnosed with organized cryptogenic pneumonia, and was chronically immunosuppressed with methylprednisolone (32 mg/day). Due to the corticotherapy, he developed type 2 diabetes with poor metabolic control despite treatment with insulin and oral hypoglycemic agents. At admission, the patient had glycosylated hemoglobin levels of 10%.

He was treated with antibiotherapy with amoxicillin/clavulanate and anticoagulation with enoxaparin, and he had a good initial clinical response. He was discharged on day four.

On the seventh day of therapy, the patient returned to the emergency department with marked pain on the right lower limb, weakness, and upper leg edema. There was no history of trauma, surgery or manipulation of the limb.

**Figure 1:**
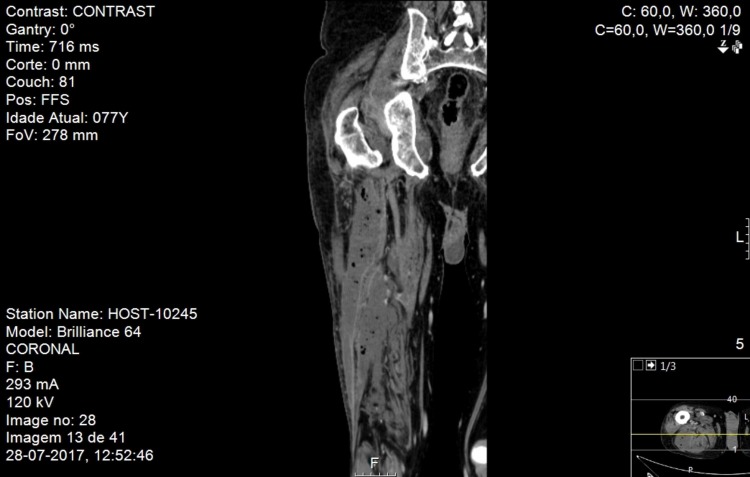
Right lower limb CT scan, frontal plane Right lower limb computerized tomography scan, frontal plane, revealing a gas collection in the region of the right iliacus muscle

**Figure 2:**
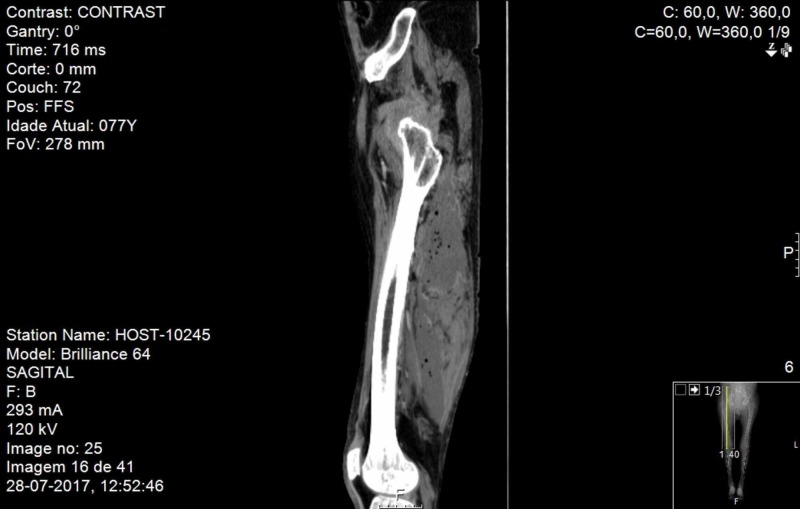
Right lower limb CT scan, sagittal plane Right lower limb computerized tomography scan, sagittal plane, revealing a gas collection in the region of the right iliacus muscle

He presented no fever, and we noted edema and hyperesthesia of the posterior region of the upper thigh. His blood pressure and heart rate were within reference ranges, with normal findings on lung auscultation. Laboratory results showed normocytic normochromic anemia (hemoglobin level was 102 g/L), leukocytosis (17.71×10^9^/L) with neutrophilia at 92.1%, and elevated C-reactive protein levels (2447.62 nmol/L; reference range, < 47.62 nmol/L). His blood lactate was 1.78 mmol/L (reference range, <2 nmol/L) Additionally, he had mild hyponatremia of 131 mmol/L. We isolated *Nocardia cyriacigeorgica* from the initial blood cultures from the first admission (matrix-assisted laser desorption/ionization-time of flight (MALDI-TOF) mass spectrometry method). A computed tomography (CT) scan of his lower limb revealed a gas collection in the region of the right iliacus muscle with involvement of the gluteal and obturator muscles upwardly and on the supragenicular plane inferiorly (Figures 1, 2). It showed luminal thrombosis of the femoral veins and prostate with hypodense areas suggesting the presence of abscesses.

Triple therapy with imipenem, amikacin, and cotrimoxazole was started, and the patient was submitted for emergent surgical decompression, fasciotomy, and drainage due to acute compartment syndrome. A culture of pus was positive for growth of *Nocardia cyriacigeorgica*.

**Figure 3:**
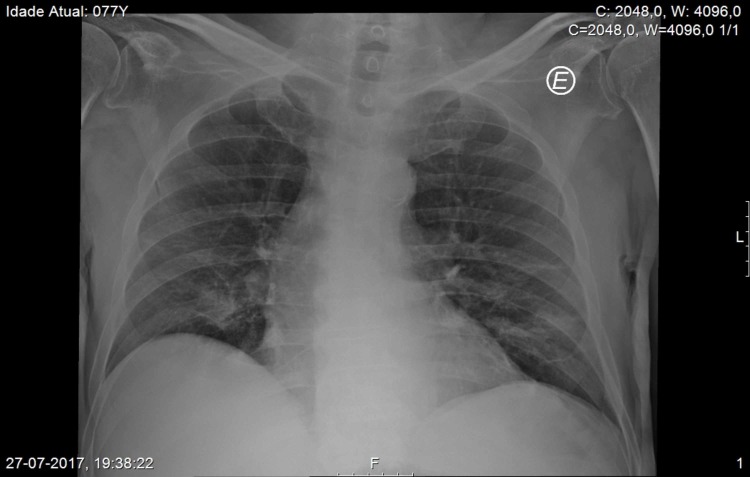
Chest X-ray, posteroanterior view Chest X-ray, posteroanterior view, on the second admission, showing residual image of organized cryptogenic pneumonia of the right lung, the patient had the year before

**Figure 4:**
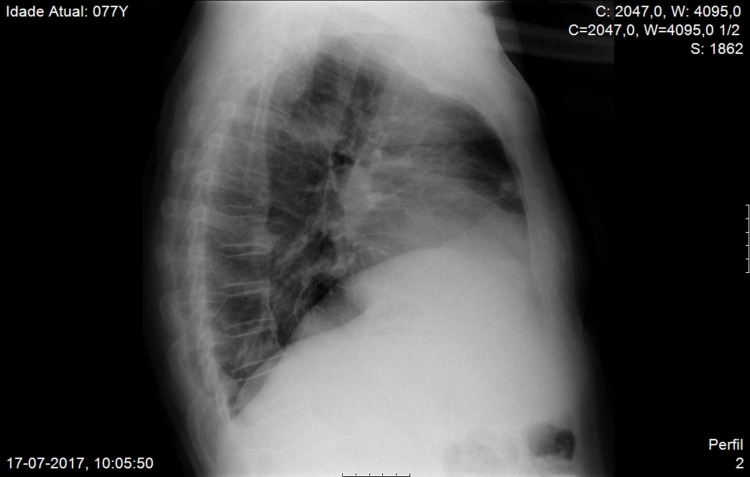
Chest X-ray, lateral view Chest X-ray, lateral view, on the first admission, with residual aspects of organized cryptogenic pneumonia of the right lung the patient had the year before

**Figure 5:**
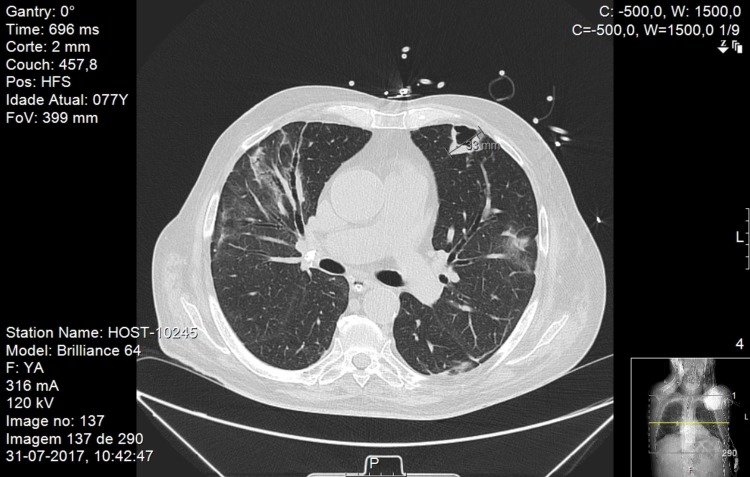
Thoracic CT scan Thoracic computerized tomography scan with cavitation with 3,3 x 3,2 cm on the anterior area of the left upper lobe

With chest x-ray at both admissions showing residual aspects of past pneumonia on the right lung, the thoracic CT scan showed cavitation with 3.3 cm x 3.2 cm on the anterior area of the left upper lobe (Figures 3-5). 

A head CT scan showed no evidence of abscess.

The patient responded well to treatment, and he was discharged from the hospital after 30 days of intravenous therapy. He continues to recuperate after five months of the initial admission, and he is continuing treatment with oral cotrimoxazole for a planned duration of one year.

## Discussion

The incidence of nocardiosis is increasing due to the increase in the number of immunocompromised patients. Recent molecular analyses have allowed for easy and accurate identification and clarification of the taxonomy of the *Nocardia* genus [8]. The geographic distribution of *Nocardia *species is very different worldwide, but a recent study in Spain identified *Nocardia cyriacigeorgica* as the most prevalent species to cause nocardiosis in that country [11]. Our country, Portugal, is a neighbor to Spain and shares many commonalities.

Corticosteroids are the most important risk factor fornocardiosis, but comorbidities like chronic obstructive pulmonary disease and bronchiectasis are also determinants for pulmonary nocardiosis [7,8].

The cutaneous disease usually occurs with local direct inoculation secondary to trauma, surgery, a vascular catheter or an insect bite [6]. However, the disease can also be secondary to hematogenous dissemination. Our patient was on corticotherapy for as long as one year. After the identification of the agent causing the disease, and given the patient had no history of trauma or manipulation in the lower limb, it was imperative to search for other lesions. Despite no evidence in the thoracic x-ray, cavitation appeared in the lung CT scan, and prostatic abscesses were also found. We theorize the primary source of infection was the lung in our patient given he had no diagnosed pulmonary comorbidity but had had pneumonia requiring admission one year prior.

Dissemination is considered whenever nocardial abscesses are found at two or more locations, and mortality is as high as 64% [6,12].

Our patient was started on directed therapy and underwent prompt surgery to perform source control: two therapeutic measures pivotal to the success of treatment and the complete restoration of the patient’s health. Our patient maintains prolonged therapy with cotrimoxazole; data suggest that strains in the Iberian Peninsula are susceptible to this antibiotic [11].

## Conclusions

This case illustrates the complexity and severity of infection in the immunosuppressed patient. The details of this case encourage a meticulous approach in similar patients. *Nocardia* infections will become more frequent in the future, and sulfonamides and bacteriostatic antibiotics are still first-line therapy.
